# Acetobacter Biofilm: Electronic Characterization and Reactive Transduction of Pressure

**DOI:** 10.1021/acsbiomaterials.0c01804

**Published:** 2021-03-29

**Authors:** Alessandro Chiolerio, Andrew Adamatzky

**Affiliations:** † Center for Sustainable Future Technologies, Istituto Italiano di Tecnologia, Via Livorno 60, Torino 10144, Italy; ‡ Unconventional Computing Laboratory, University of the West of England, Coldharbour Lane, Bristol BS16 1QY, United Kingdom

**Keywords:** bacterial cellulose, biomaterials, sensing, sensorial fusion, soft robotics

## Abstract

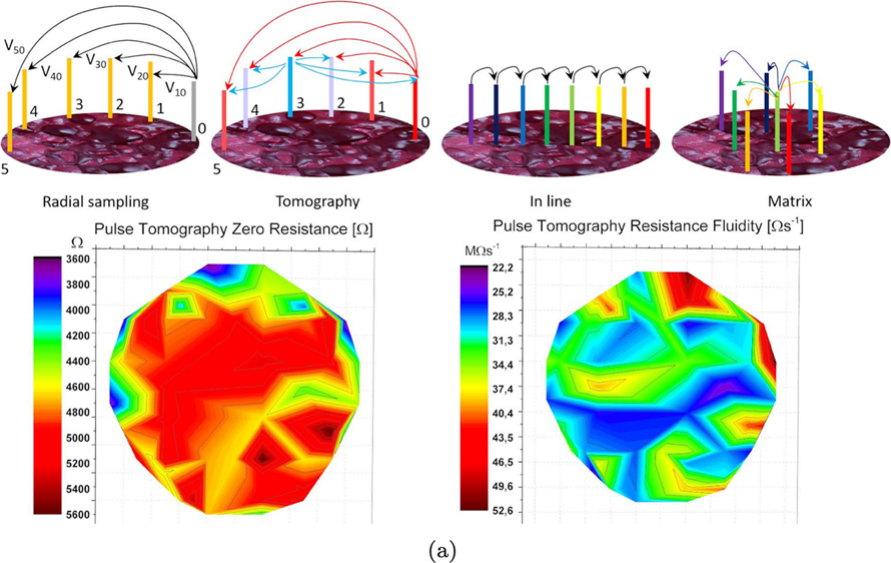

The bacterial skin studied here is a several centimeter-wide colony of *Acetobacter aceti* living on a cellulose-based hydrogel. We demonstrate that the colony exhibits trains of spikes of extracellular electrical potential, with amplitudes of the spikes varying from 1 to 17 mV. The bacterial pad responds to mechanical stimulation with distinctive changes in its electrical activity. While studying the passive electrical properties of the bacterial pad, we found that the pad provides an open-circuit voltage drop (between 7 and 25 mV) and a small short-circuit current (1.5–4 nA). We also observed by pulsed tomography and spatially resolved impedance spectroscopy that the conduction occurs along preferential paths, with the peculiar side-effect of having a higher resistance between closer electrodes. We speculate that the Acetobacter biofilms could be utilized in the development of living skin for soft robots: such skin will act as an electrochemical battery and a reactive tactile sensor. It could even be used for wearable devices.

## 1 Introduction

Flexible artificial skins^1−3^ represent a fast-growing research field, involving several disciplines under the umbrella of novel electronics and materials science and, particularly in the last few years, also their biology and sustainability. Artificial skins are typically soft, flexible, and eventually stretchable materials enabling pressure/tactile sensing with a certain spatiotemporal resolution^4−7^ and allow, for example, low-level perception.^8,9^ A well-known architecture is based on thin-film transistors connected to pressure sensors,^10^ nanocomposite soft thin foils, incorporating conductive fillers such as carbon nanotubes,^11,12^ chemically processed tungsten films,^13^ multilayered graphene layers,^14^ platinum ribbons,^3^ silver electrodes,^15^ digitally printed hybrid electrodes,^16^ piezoresistive sensors,^17^ piezoelectric sensors,^18^ random network-based conductive polymer-filled soft channels,^19^ and many other solutions.

One of the most impacting issues regarding both electronic devices and nanocomposite materials is represented by their poor (in most cases null) capability to self-repair and grow, to self-organize and adapt to changing environmental conditions, and to be sustainable since the synthesis. There are a few exceptions to this, for example, very recently, a bioinspired hydrogel was developed, providing self-healing ability, stretchability, 3D printability, and electrical conductivity.^20^

Biological, living organisms represent a particular choice in this direction that enables addressing all of the previous issues, further opening new exciting horizons for what concerns signal processing, data fusion, and multisensing capabilities. Living organisms could perfectly match unconventional living architectures^21^ and soft and self-growing robots.^22−25^ For example, tactile, color sensors were fabricated using slime mold *Physarum polycephalum*, which are unicellular macroscopic fungi that process their nutrients through a complex network that preserve their adaptability and regeneration capabilities.^26−28^ Fungal electrical activity was also studied and demonstrated to be in line with typical sensing and computational requirements.^29−31^ Fungi have been proposed as human skin surrogates for wound healing purposes.^32−37^ More recently, we have demonstrated a reactive fungal wearable made out of *Pleurotus ostreatus* mycelium.^38^

The particular substrate we have selected and studied is the mother of vinegar, a colony of *Acetobacter aceti* living on a cellulose-based hydrogel that typically floats on top of wine and converts ethanol into acetic acid with the help of oxygen.^39^ Acetobacter is one among a group of bacteria that are able to synthesize cellulose, including *Azotobacter*, *Rhizobium*, *Pseudomonas*, *Salmonella*, *Alcaligenes,* and others.^40^ The main difference in comparison to plant cellulose is that the bacterial pellicle is composed of more pure cellulose, that does not contain any hemicellulose or lignin, and has smaller fibers, less than 100 nm diameter, that bear higher mechanical stresses^41^ and has been proposed for several functional and structural applications, including engineering of the biosynthesis processes^42^ and genetic engineering to enhance production.^43^ To the best of our knowledge, this is the first complete assessment of a living bacterial colony’s electrical properties, in view of potential functional artificial skin applications. For the time being, we have focused our study on the electronic collective properties of the colony and found several non-trivial features. More advanced experiments are under development and will be discussed in future papers. *Acetobacter* was made to colonize a cotton glove on its entire surface; its electrical properties were assessed to interpret the electrical spiking of the colony and associate that to the pressure stimuli applied on the glove. The colony is kept wet and alive with a nourishing broth, while for practical applications, other bacterial strands have to be exploited, granting their operation with lower amounts of moisture.

The paper is structured as follows: we describe the methods for electrical activity recording and dc, ac, impedance, and pulse tomography characterizations in Section 2. Patterns of electrical activity and measured electronic properties are analyzed and discussed in Section 3. Final remarks in a wider context and conclusions are drawn and future directions are suggested in Section 3.5.

## 2 Methods

*Acetobacter* biofilms are produced by positioning an already formed pellicle of *Acetobacter* in a glass 70 mm in diameter, filled with 100 mL of red wine (Barbera, 14% alcohol volume content, <0.2% residual sugar content) as a nutrient, and keeping it in a bath at a constant temperature of 25 °C for 1 week. The particular strand of *Acetobacter aceti* we have used comes from an artigianal production of red vinegar, performed in Vigliano d’Asti, Italy by one of the authors and cultivated (i.e., preserved alive without replication) for approximately 50 years. A second pellicle develops as the surnatant, floating at the interface between liquid and air, growing in the same circular shape and diameter as the glass. The homogeneity of the pad, as put in evidence by the electronic characterization techniques, is quite good. Thickness of the membranes ranges between fractions of mm up to some cm, as a function of the incubation temperature of the bath and incubation time. In our case, the thickness was 5 mm.

**Figure 1:**
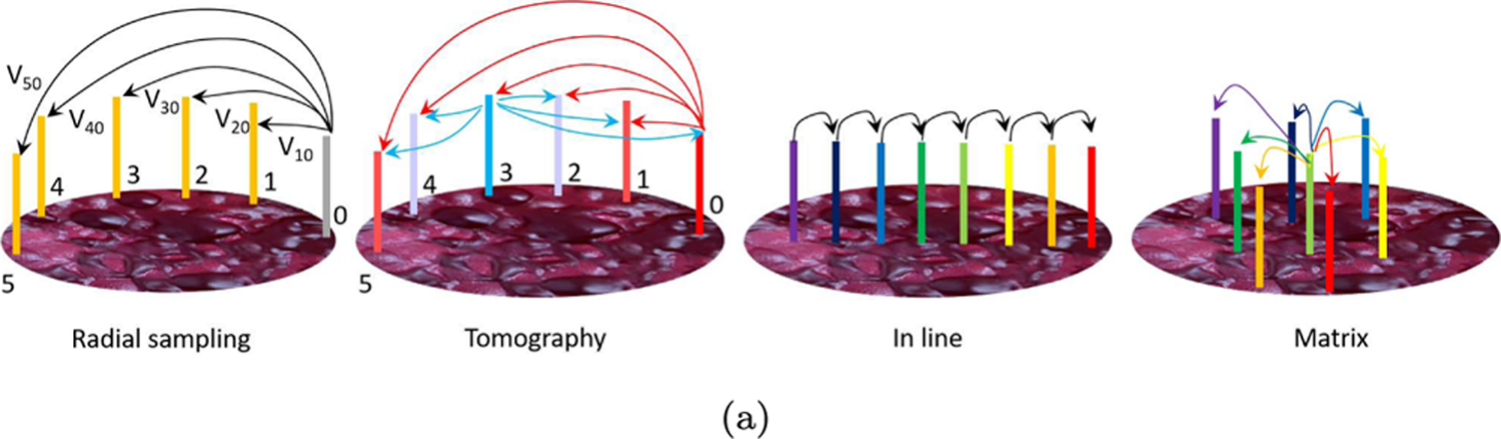
Scheme showing the four electrode arrangements used in the paper to characterize electrical properties. From left to right: radial sampling: it is used for IV and impedance properties; the reference electrode “0” is kept fixed and all the other 15 electrodes are used as probes to provide a radial resolution, so that the final amount of measurements is *n* – 1 if *n* is the number of electrodes. Tomography: similar to radial sampling, but each electrode position is used not only as a probe but also as a reference, so that the final number of measurements is *n*(*n* – 1). In-line sampling: it is used to monitor the progress of a spike along a specific direction. Since each electrode works in a differential mode, the final number of measurements is *n*/2. Matrix sampling: used again to monitor the progress of spiking activity with a bidimensional resolution; the final number of measurements is *n*/2.

The passive electromagnetic properties of the *Acetobacter* skin were measured as follows. Two needle electrodes (MN4022D10S subdermal electrodes from SPES MEDICA SRL Via Buccari 21 16153 GENOVA, Italy, 0.4 mm diameter, 22 mm in length were used to contact the skin along the entire circumference, placing them in 16 loci, positioned at an angular distance of 22.5° (see Figure 1, far left). The electrode placed at, say, 0° was used as a reference, measurements were taken in the clockwise direction, using the couple (0–22.5), (0–45), and so on. During measurements, the skin was placed in a Petri dish without a liquid matrix; the longest dc measurements took approximately 3 h and evaporation and drying of the skin significantly affected recordings. Therefore, a linear correction of the drying-induced drift was always implemented, both on IV curves and on relaxation measurements, on the basis of repetitions of recordings taken from the same couple of electrodes at the beginning and at the end of the run. Impedance measurements were much faster (<1 h) and no significant evaporation or drying was observed; consequently, no drift occurred. A Keithley 2635A multimeter was used for dc characterization (IV curves in the range ±20 mV) and relaxation measurements (50 mV was kept on one electrode, and the current was measured as a function of time) and an Agilent E4980A precision LCR meter for ac characterization (range from 20 Hz up to 2 MHz, 20 mV_RMS_). Pulse tomography was performed using eight electrodes placed at an angular distance of 45^°^. The pulse was 50 mV in amplitude and 50 μs in duration; the instrument, a Keithley SCS 4200, was allowed to acquire 500 pulses to reduce collection noise. Each couple of electrodes was measured twice, using as reference both contacts, and data were inverted to obtain a tomography of the impedance of the *Acetobacter* skin (see Figure 1, middle left).

The electrical activity of the skin was measured as follows. We used iridium-coated stainless steel subdermal needle electrodes (Spes Medica S.r.l., Italy) with twisted cables. The pairs of electrodes were inserted in the *Acetobacter* pellicle. In each pair, we recorded a difference in electrical potential between the electrodes (see Figure 1, middle right and far right). We used an ADC-24 (Pico Technology, UK) high-resolution data logger with a 24-bit analog to digital converter, galvanic isolation, and software-selectable sample rates. We recorded electrical activity with a frequency of one sample per second. We set the acquisition voltage range to 156 mV with an offset accuracy of 9 μV to maintain a gain error of 0.1%. For mechanical stimulation, we used an 80 g nylon cylinder, the contact area with the colony skin was ca. 7 cm disc, which is ca. 1–2 mm thin.

## 3 Results and Discussion

### 3.1 *IV* Characterization

**Figure 2:**
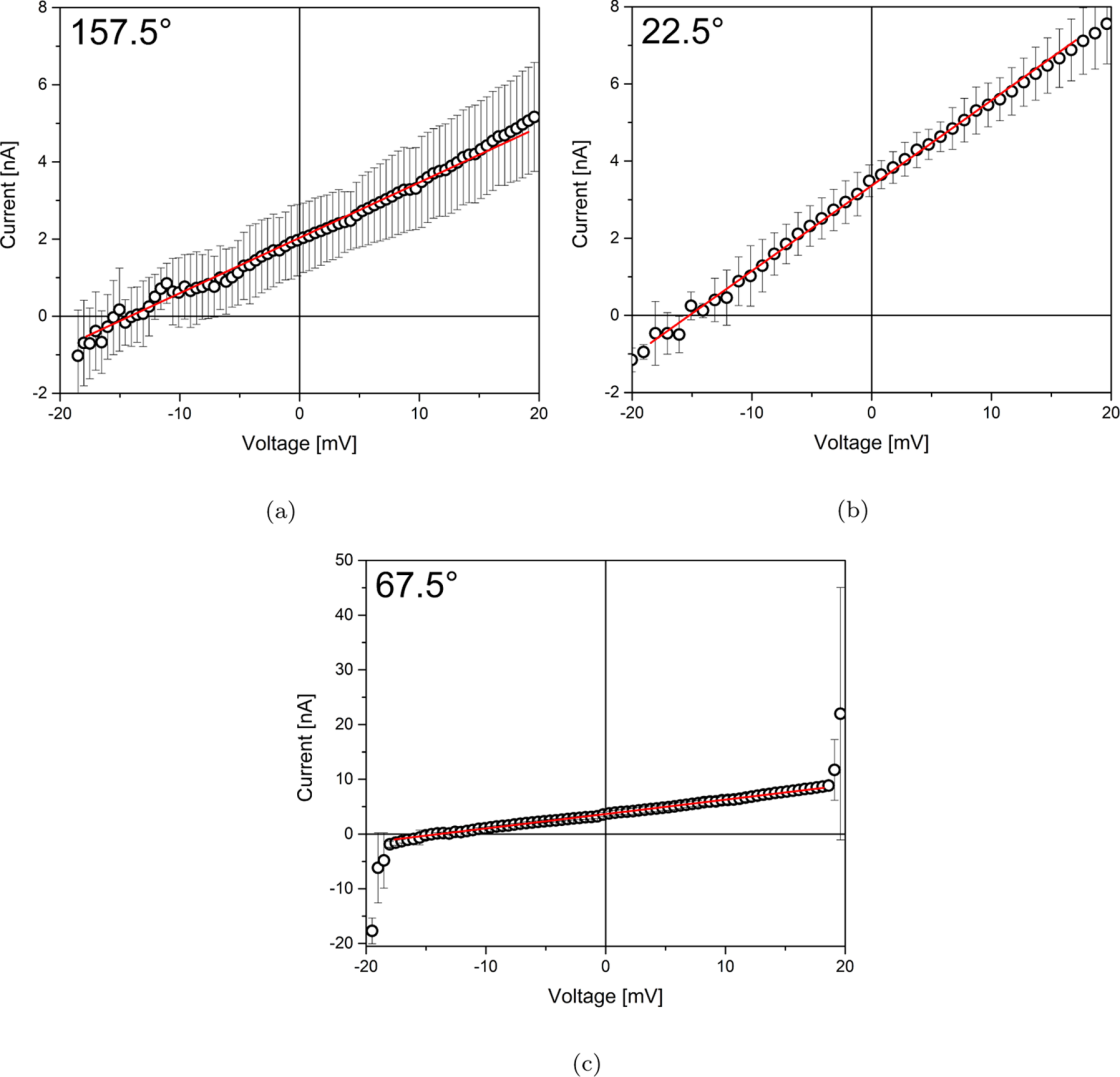
(a) *IV* curve measured at 157.5°. (b) *IV* curve measured at 22.5°. (c) *IV* curve measured at 67.5°.

*IV* curves show three typical trends: an approximately linear trend with a rather high noise (Figure 2a), a marked nonlinearity in quadrant III (for negative potentials), followed by a stable and linear part (Figure 2b), or a marked nonlinearity in both quadrants III and I plus a linear and extremely stable part in between (Figure 2c). The difference in macroscopic behavior is most probably due to the underlying connection scheme between cellulose fibers: if the connection is straightforward, charge carriers propagate as in an Ohmic conductor. If the connection is indirect and occurs through the superposition of separate fibers, the contact resistances build up a nonlinear component to the *IV* curves. Intermediate responses, such as those showing only a slightly nonlinear curve and/or a noisy structure, indicate that the number of indirect jumps controls the ultimate characteristics. In the literature, typical results show quite smaller resistances, in the range of a few tens of Ωs up to a hundred Ω, but bacterial cellulose is applied as an electrolyte and therefore prepared by soaking into highly concentrated ionic solutions, such as KOH and KI,^44^ so it is difficult to compare these with our case. Since the bacterial cellulose hosts a living colony, which is what we want to characterize, to study phenomena such as spontaneous spiking, we did not make a blind test on sterilized cellulose.

**Figure 3:**
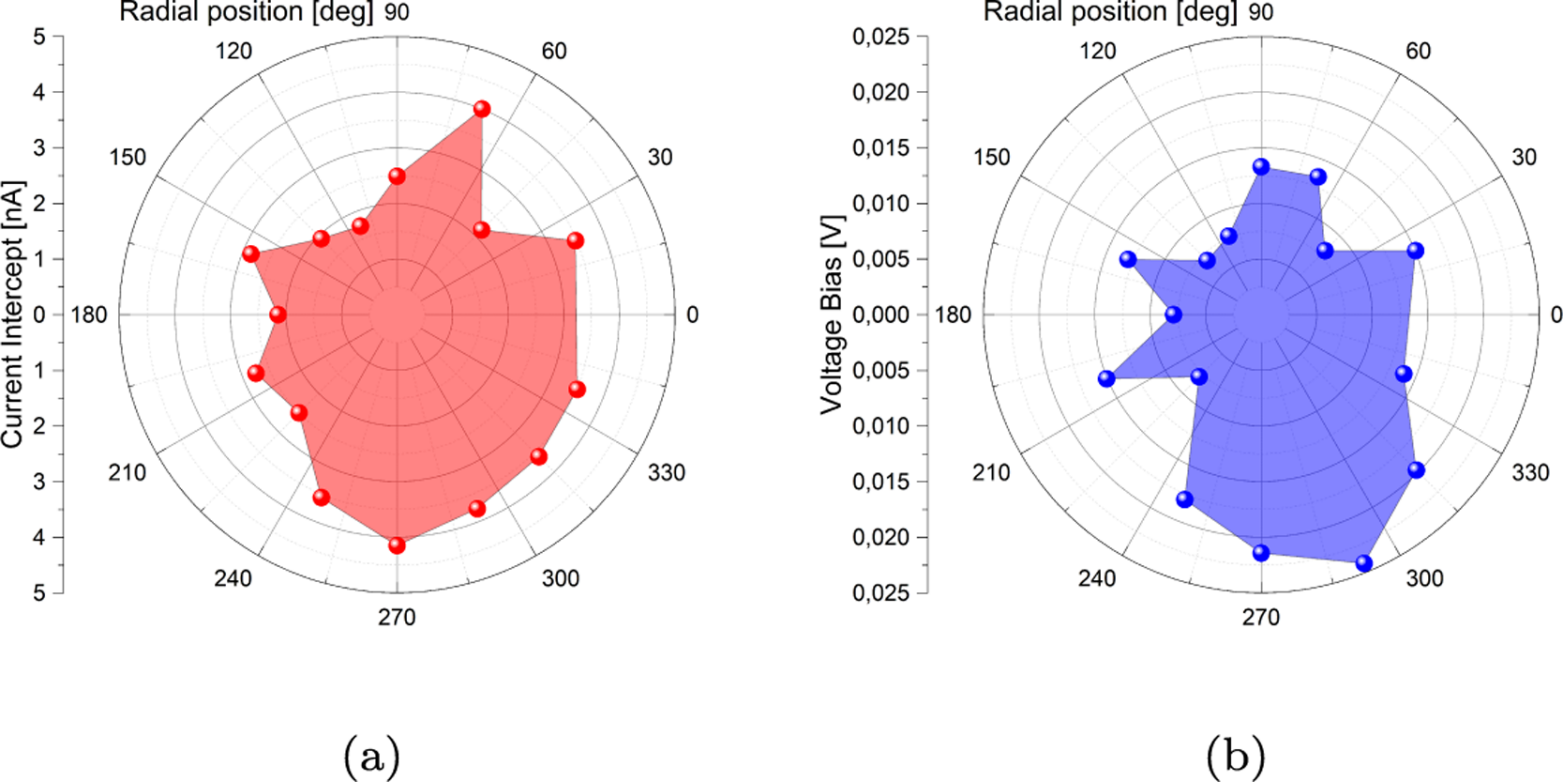
(a) Polar plot of the short-circuit current distribution on the *Acetobacter* circular skin. (b) Polar plot of the open-circuit voltage distribution on the *Acetobacter* circular skin.

By fitting the linear part we derived the open-circuit voltage (potential measured when no current flows) and the short-circuit current (current measured when no potential is applied), both always nonzero, and plotted them against the position of the active electrode for measurements, in polar coordinates (Figure 3a). This result means that the disk-shaped biofilm is not entirely homogeneous from an electrical point of view. By using the electrode at 0° as reference (hence polarized at 0 V), the measured current is always positive in the range [−10, +20 mV], so the positive ions responsible for electrical conduction move toward it. In order to reverse the current sign, it is necessary to add an extra potential, below −10 mV. This can be interpreted in terms of a galvanic phenomenon, comprised between 7 and 25 mV, depending on the area under test (Figure 3b).

**Figure 4:**
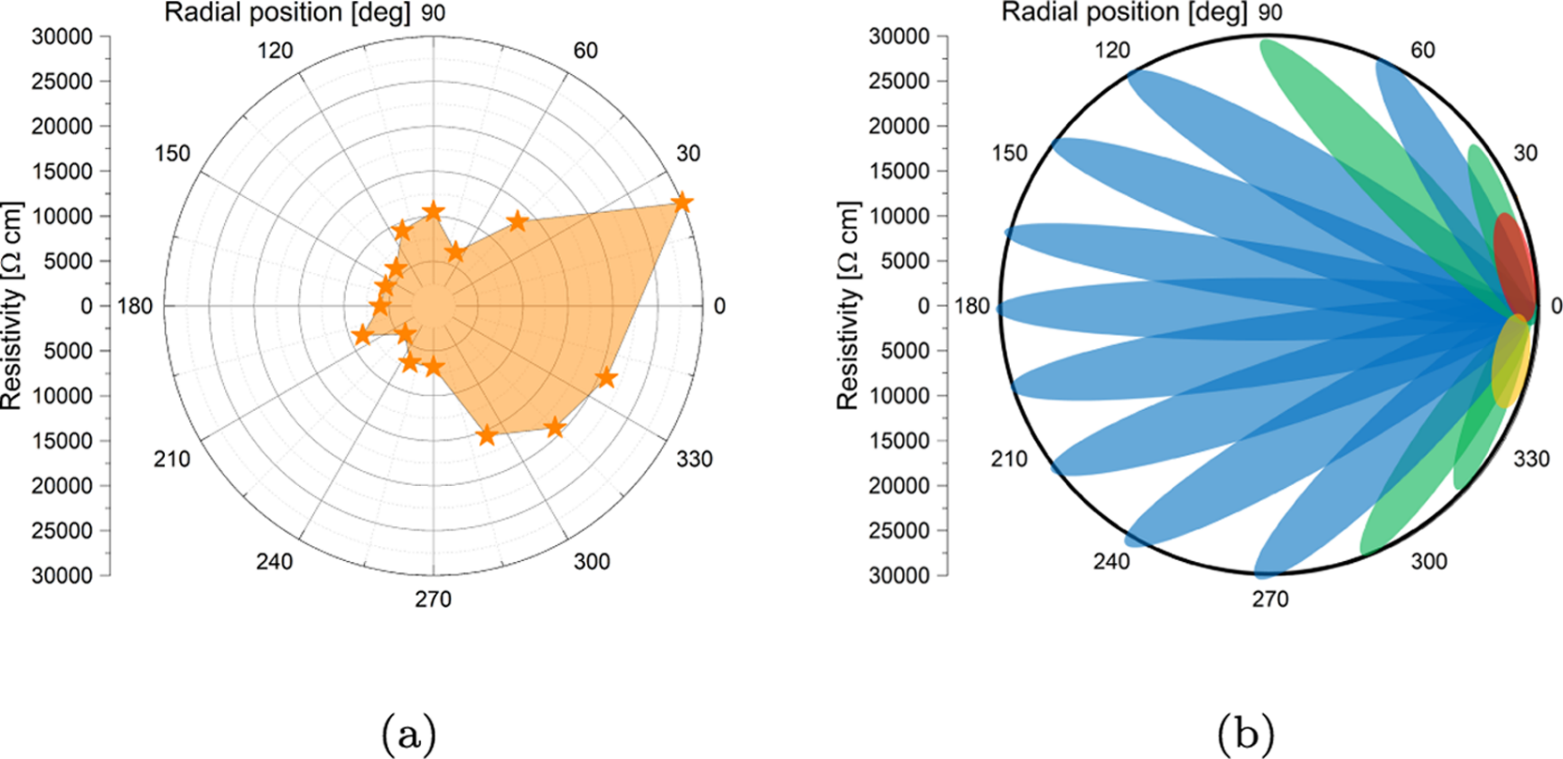
(a) Resistivity extracted from *IV* curves, represented in a polar graph. (b) Leaf graph to reconstruct morphological features in the biofilm based on the resistivity data.

The biofilm, without any specific means to collect electronic charge and without specific nutrients added in the matrix, provides a maximum of 100 pW of power per electrode couple. Another peculiar feature that can be extracted by the linear fit is resistivity. Seen in polar coordinates (Figure 4a), one can notice that the highest values of resistivity are reached in the colony portion between closer electrode couples. This aspect is counter-intuitive but might be explained by studying the cellulose network developed to support the colony, as all electrical signals move along cellulose fibers and can be supported in their movement by oriented fibers. A particular texture, conceived to efficiently propagate information from the core to the periphery, could better connect distant points rather than closer ones. The leaf diagram has been prepared on the basis of numerical data, assigning a color code to each leaf, positioned in the space between the reference (0^°^) electrode and the active one (Figure 4b). The leaf diagram is constructed according to the following procedure: each leaf represents a specific feature measured across the electrodes placed at its ends, keeping the orientation (e.g., between 90 and 0°); the leaf is colored with a partially transparent filling, to allow appreciation of superposition, and the tint is assigned according to the magnitude of the specific property shown, going through blue (minimum), green, yellow, orange, and red (maximum). In case a dishomogeneity of conduction properties is present, the leaf diagram allows us to immediately locate the anomalies. The necessity to build a leaf graph is that this representation is more directly connected to the morphology of the system, though the information content is the same as in the polar plot. But here, referring to the color of each leaf, we can immediately locate the anomaly, if any, or the symmetry of the property measured.

### 3.2 Relaxation Measurements

Relaxation measurements require a recording of at least 300 s to capture the asymptotic behavior. Drift-subtracted curves were fitted using the Farazdaghi–Harris rational model, according to eq 1
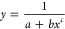
1

**Figure 5:**
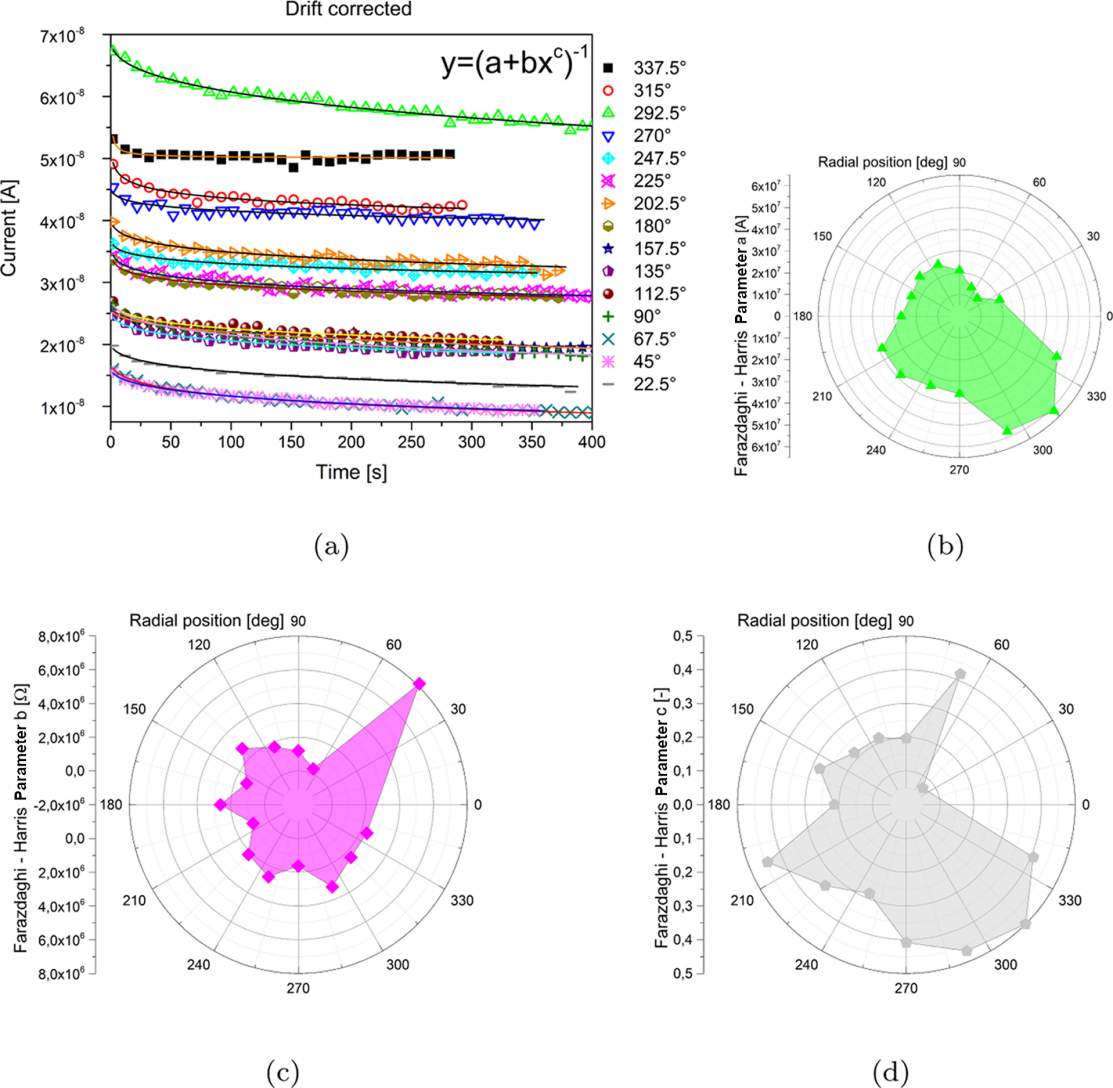
(a) Relaxation curves recorded from all electrode couples, as a function of the aperture angle between sample and probe electrodes (1 point for every 5 shown for clarity). (b) Polar representation of the distribution of values taken by parameter *a* from the model in eq 1. (c) Polar representation of the distribution of values taken by parameter *b* from the model in eq 1. (d) Polar representation of the distribution of values taken by parameter *c* from the model in eq 1.

The quality of the fit can be appreciated from Figure 5.

**Figure 6:**
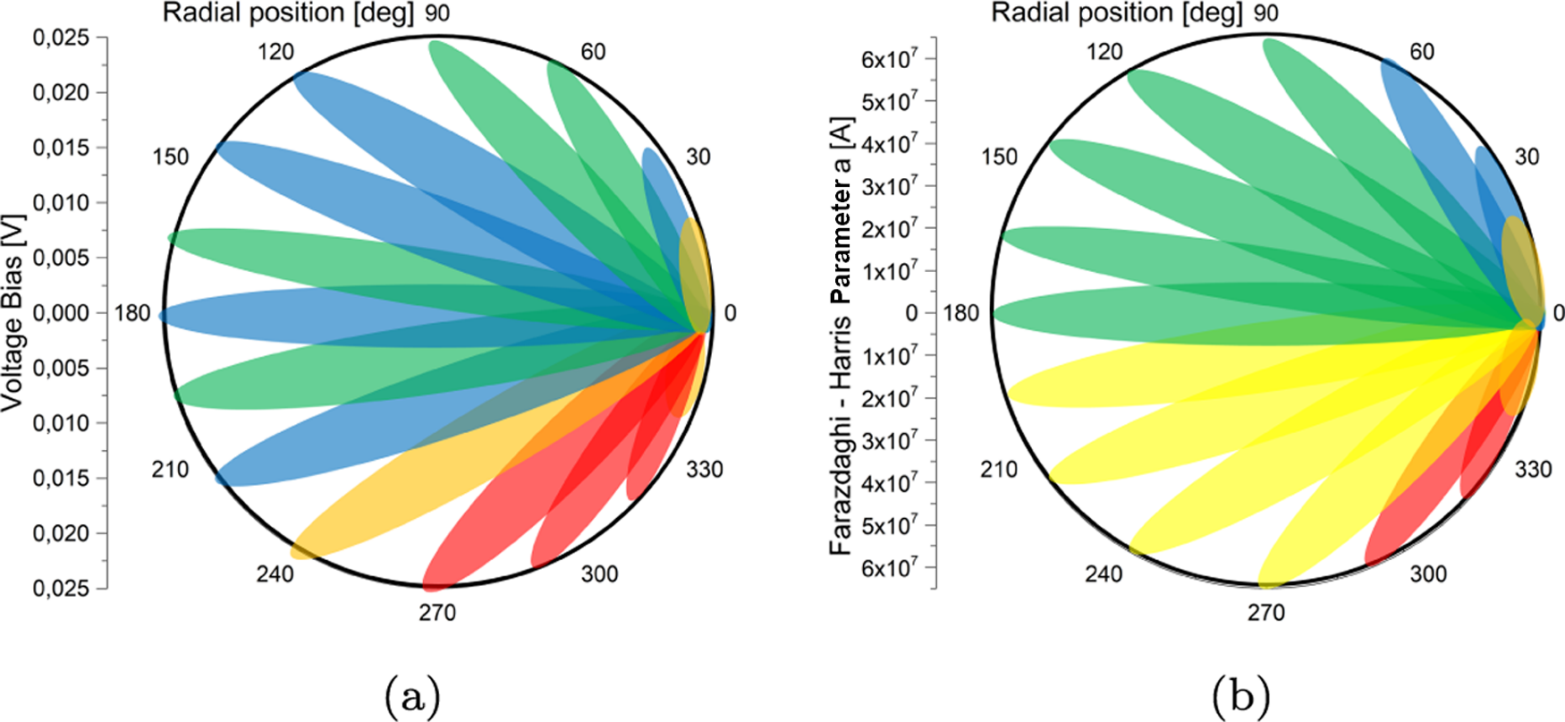
(a) Leaf graph to reconstruct morphological features in the biofilm based on the open-circuit voltage data. (b) Leaf graph to reconstruct morphological features in the biofilm based on the *a* electrical relaxation fit parameter.

Intuitively, fit parameters can be associated with an asymptotic current flow (a), a resistance (b), and a non-dimensional scale parameter (c). The polar representation of such parameters is shown in Figure 5b–d. We can see the anisotropy axis emerging from the diagrams, oriented along the 150/330^°^ diameter (wide green and grey areas) and the 60/240^°^ diameter (magenta and grey spike). Morphological features have been detected exploiting some of the measured conduction properties, in particular the open-circuit voltage and the Farazdaghi–Harris parameter *a* of the relaxation measurements, Figure 6. Both such parameters show that the *Acetobacter* pad features a zone comprised between 202.5/240 and 337.5^°^ where both the open-circuit voltage and the *a* parameter feature higher values. The projective nature of the measurements performed allows us to roughly locate the anomaly that produces higher voltages and currents, in other words, where the electricity generation occurs, but not to deduce its shape and extension with sufficient accuracy. Other tomographic techniques could be used but should be implemented preserving the moisture content of the skin, to avoid drying, considering the time needed to collect data (approximately 1 h per reference electrode, times 16 electrodes). Nevertheless, we succeeded in performing a fast pulse measure and collected data from each of the sample electrodes, highlighting the nonhomogeneous structure inside the network. A percolating branch is seen, having less than half the conductance of the surrounding areas.

### 3.3 Impedance Spectroscopy with Angular Resolution

**Figure 7:**
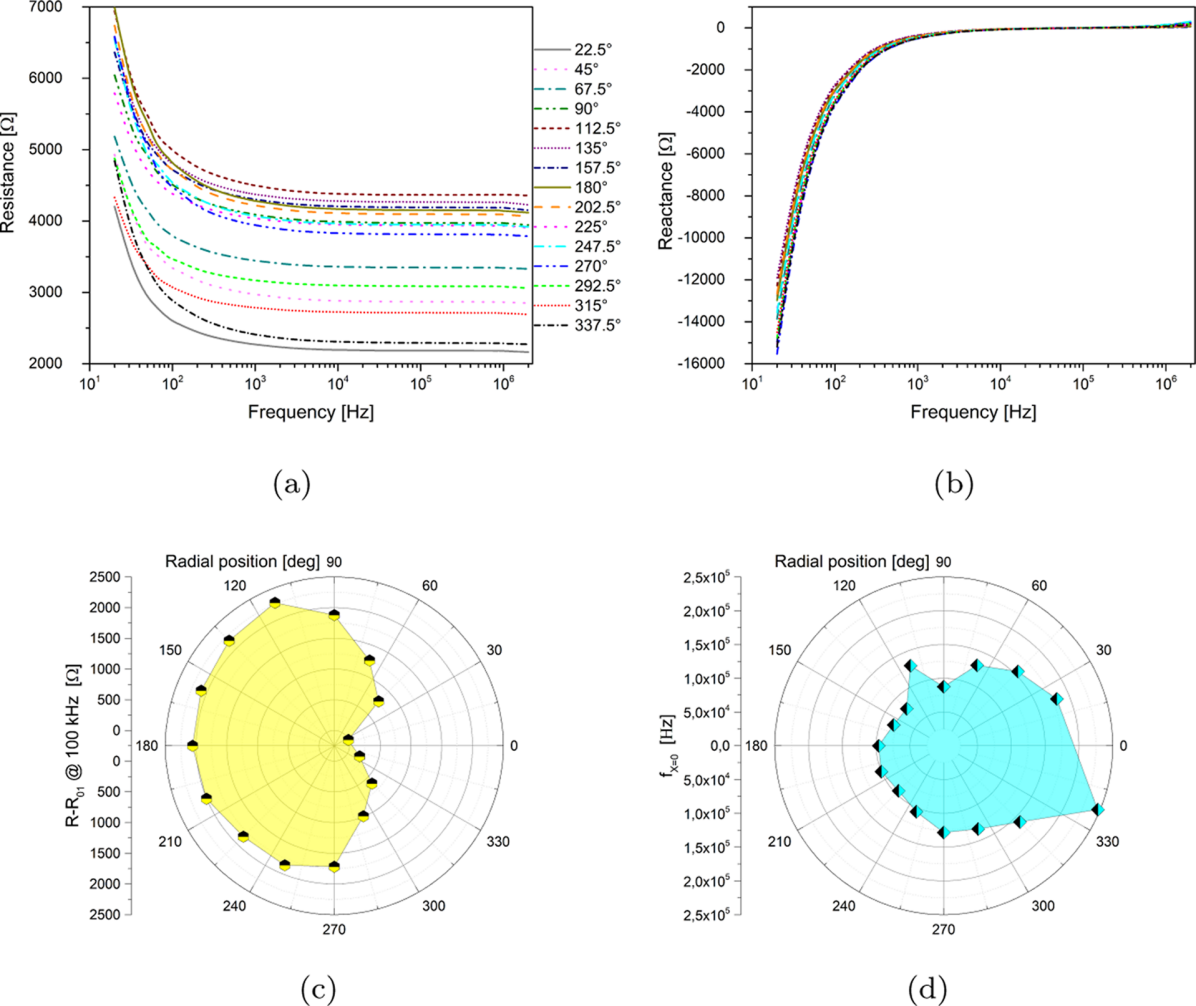
(a) Resistance as a function of frequency for all the electrode couples. (b) Reactance as a function of frequency for all the electrode couples. (c) Differential resistance at a frequency of 100 kHz in polar coordinates. (d) Frequency of transition between capacitive and inductive behavior in polar coordinates.

Impedance measurements are shown in Figure 7. The real part of impedance (resistance) shows a low-frequency capacitive-like trend up to 1 kHz, followed by an almost linear portion ranging up to the MHz range. The imaginary part of impedance (reactance) is negative up to 1 kHz, confirming a capacitive response. By carefully observing the curves in Figure 7a,b as a parameter of the aperture angle between the two electrodes, the reader will notice that symmetric curves (e.g., 22.5 and 337.5, 45 and 315^°^, and so forth) are very close. This is the first proof of the impedance homogeneity of the biofilm. Relevant parameters that can be extracted are the differential resistance (7c), normalized with respect to one channel (01), and the frequency where reactance crosses the zero axis (7d), transitioning between the capacitive and a slightly inductive behavior. Such parameters have been plotted in polar coordinates. The differential resistance polar plot is a perfect example of a purely morphological feature: the two lobes that can be seen, and the symmetry of the parameter with respect to the radial coordinate, mean that the property is mostly influenced by the distance between the reference and active electrodes used for recordings, reaching the maximum at 180^°^ and the minimum at 0^°^. In a similar manner, the frequency parameter, witnessing transition between capacitive and inductive responses, is higher when the associated values of capacitance are smaller (i.e., when contacts are closer, they transition to inductive behavior “earlier”) and the pattern in the polar graph is almost perfectly oriented parallel to the *x* axis. The signal recorded can be different because when doing impedance spectroscopy, charges are put in oscillation and might therefore explore more than one preferential path. As the underlying connection scheme is not symmetrical but depends on the cellulose structure, each probe excites different paths near the area where it is positioned.

### 3.4 Pulse Tomography

**Figure 8:**
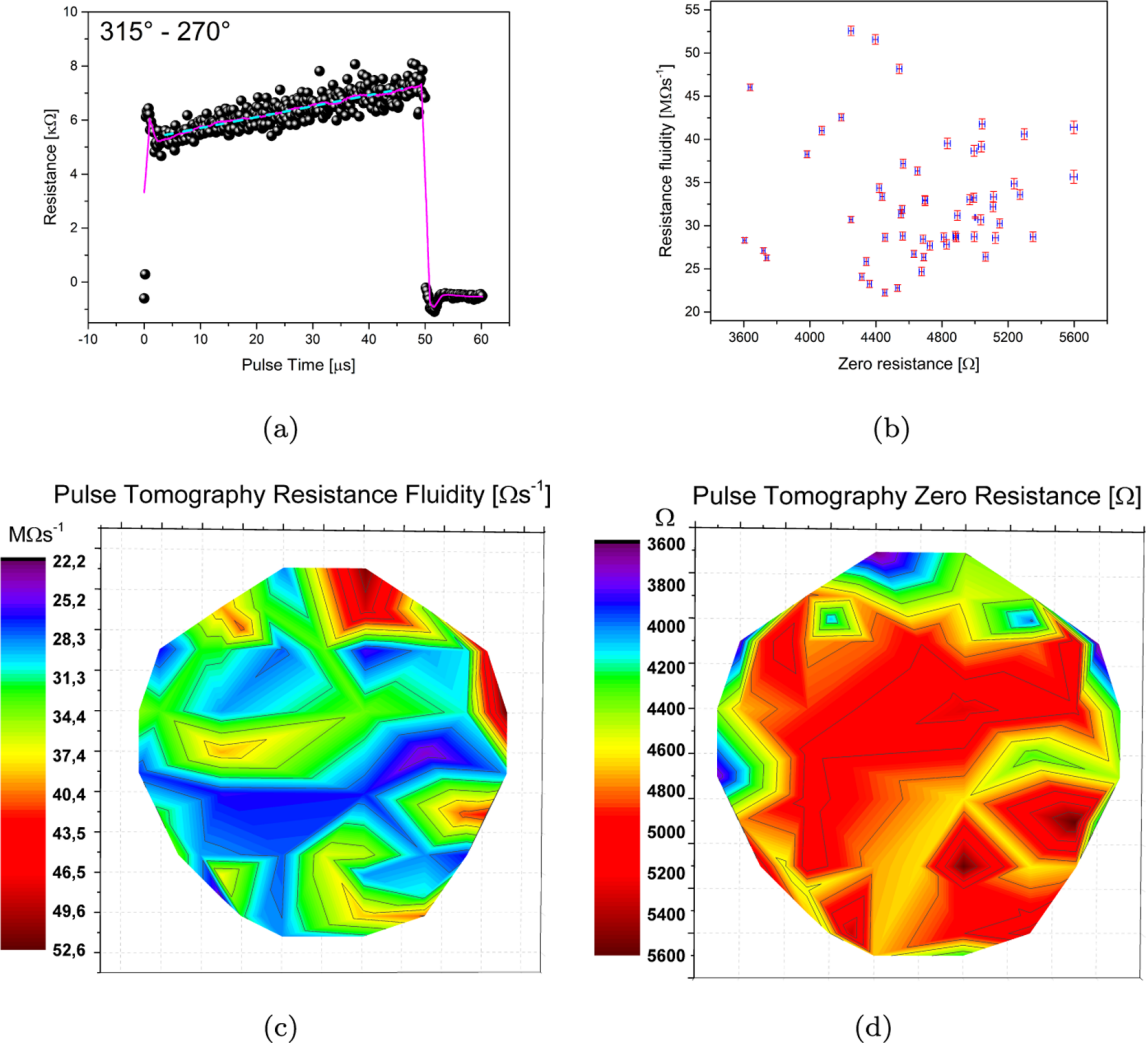
(a) Sample average resistive response to over 500 pulses subjected between electrode 14 (315^°^) and electrode 12 (270^°^), 1 point for every 10 shown for clarity; a smoothing is also shown for clarity (Savitzky–Golay first order, window of 99 points), as well as linear fit parameters. (b) Parameter space, including data extracted during the pulse tomography experiment: resistance fluidity vs zero resistance, all errors on both estimates are given. (c) Resistance fluidity extracted as the slope of the linear fit to the average response. (d) Zero resistance extracted as the intercept of the linear fit to the average response.

Pulse tomography was performed by measuring the voltage and current provided not only by the active electrode in each measurement couple but also those for the idle electrode so that real device resistance could be computed. A typical response to pulsed voltage is shown in Figure 8a, including the linear fit parameters that have been extracted, in particular the intercept (dimensions of resistance, “zero resistance”) and the slope (dimensions of resistance over time, “resistance fluidity”) shown in Figure 8b. Such parameters have been mapped on an inverted image that allows us to spot the underlying invisible structure of connections and preferential paths (Figure 8c,d). The main finding here is a very good correspondence between areas with small zero resistance and high fluidity, while a higher resistance is associated with higher inertia of the system to adapt to the pulse stimulus and therefore a lower fluidity.

### 3.5 Spiking Recordings

**Figure 9:**
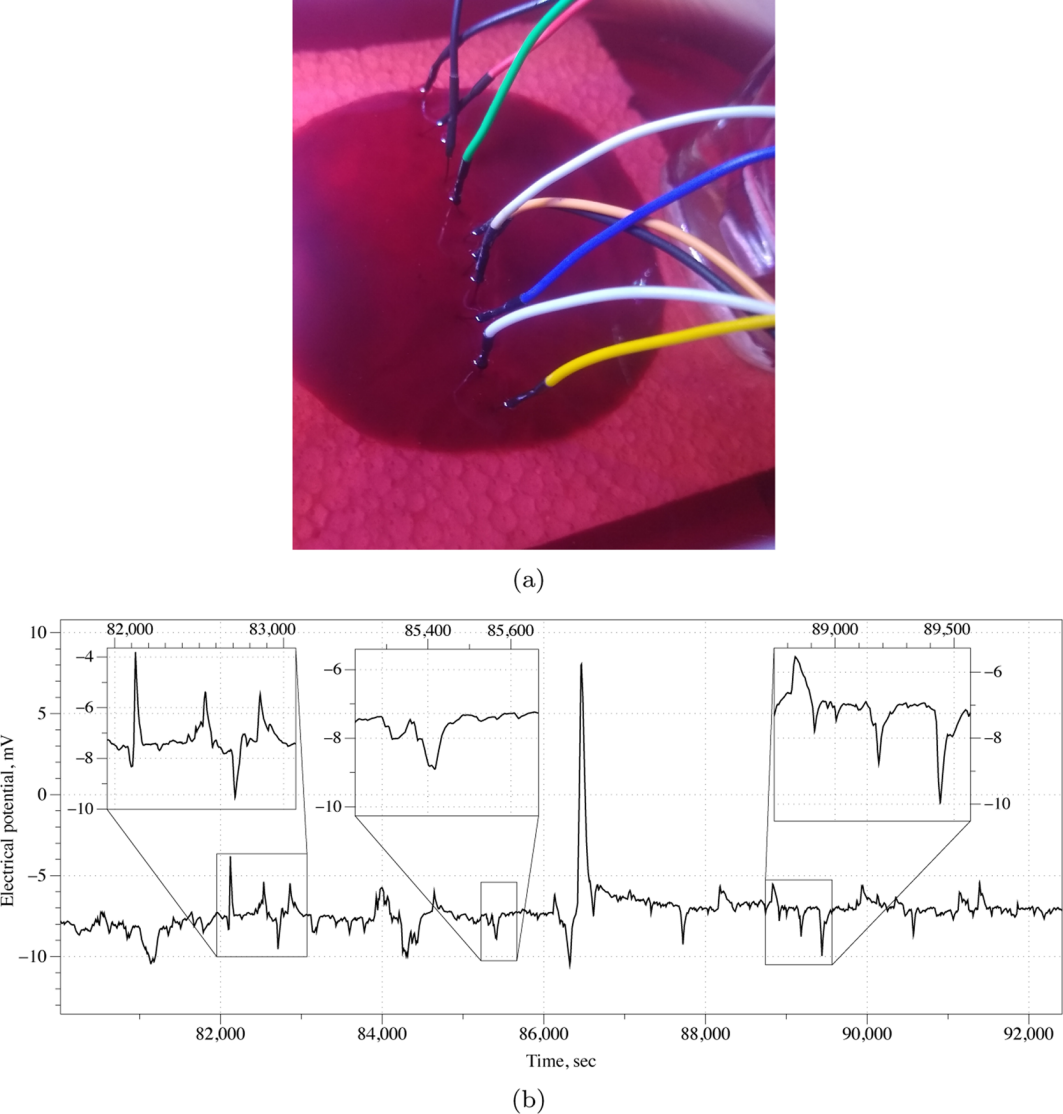
(a) Experimental setup with a linear arrangement of electrodes. (b) Example of spontaneous spiking activity of the bacterial living colony. Two trains of spikes, left and right, and one solitary spike, center, are zoomed.

Recordings of spontaneous spiking activity of the bacterial living colony are measured with two different electrode setups. One is with needles placed in-line onto a polyurethane base to keep the electrodes stable during the electrical characterization steps (Figure 9a), separated by ca. 10 mm. A second one is with needles placed arranged in a matrix 3 × 3 × 2, with a spacing of ca. 10 mm along both coordinated directions of the plan.

We observed a range of electrical potential spontaneous spiking events; an example is shown in Figure 9b. The average amplitude of spikes is 1.74 mV (median is 1.7 mV, σ = 0.98). The average width of a spike is 242.25 s (median 211.5 s, σ = 93.17). Spikes often appear in trains, 3–4 spikes per train. An average distance between spikes in a train is 516.7 s(σ = 95.2).

**Figure 10:**
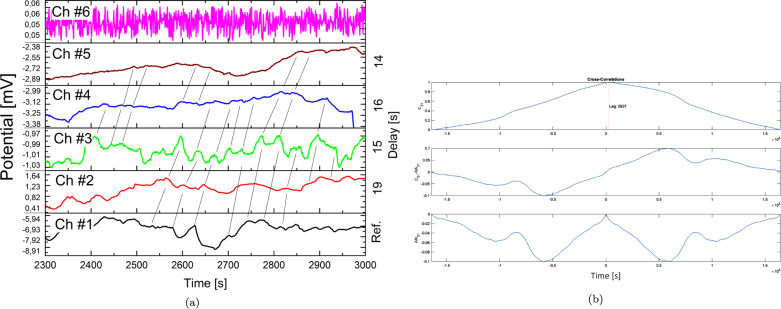
(a) Portion of recordings of spontaneous spiking activity of the bacterial living colony during a 700 s time interval, where peaks have been manually identified and followed to extract average delays in the propagation of signals. Excitatory and inhibitory functions are evident when following black lines that trace peaks. (b) Correlations between the signal on channel 2 and signal on channel 1. Top row: raw cross-correlation with an indication of the lag time corresponding to its maximum value (*x*_corr_ = 1, lag time = 29.3 s). Middle row: the same curve after computing detrend with reference to pure autocorrelation (piecewise linear, semidefined positive) showing asymmetries. Bottom row: correlation curve after computing detrend with reference to pure autocorrelation (piecewise linear, defined positive).

Recordings of spontaneous electrical potential show a similarity structure, where both excitatory and inhibitory modulations operate: a spike can be either amplified or depressed along its path crossed by the recording pairs (Figure 10a). The typical delay that spike trains require to propagate is comprised between 20 and27 s, meaning that the ultimate speed ranges between 0.37 and 0.5 mm/s. In superior animals, a spike propagates with a broad spectrum of speeds, ranging from the order of 1 m/s along the sciatic nerve axon of a frog and up to 100 m/s and beyond along the spinal motor neuron of a cat.^45^ Of course in these cases, there are (living) organs, that have been designed by evolution to support the propagation of the pulses, whose metabolism supports such propagation. In the case of a bacterial colony, cellulose is inert and can support protonic propagation.^46^ In our experiments with slime mold *P. polycephalum,* we found spikes with duration 60–120 s when recording with electrodes 10 mm apart.^47^ This might indicate that a spike velocity in slime mold is ca. 0.08–0.17 mm/s which is well in line with the velocity estimation for an *Acetobacter* biofilm. In order to quantitatively assess similarity in recordings, we have used an estimate of the deterministic correlation between two deterministic signals (Figure 10b). The true cross-correlation sequence of two jointly stationary random processes, *x*_*n*_ and *y*_*n*_, is given by
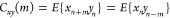
2where *n* and *m* are the indexes of experimental entries, *x* and *y* are the signals (in Figure 10*x* = 1, *y* = 2), and *E* is the expected value operator. In our case, *m* takes the meaning of the lag time: the exact amount of temporal shift of one signal that maximizes similarity between recordings. Results of correlation analyses indicate that for the linear electrode arrangement, two measures are correlated with a lag time of 31.1 and 29.3 s. For what concerns the matrix electrodes configuration, only some locations show correlation with neighboring electrodes, in particular: (1, 2), (1, 3), (4, 5), (5, 6), (5, 8), and (3, 6). The orthogonally placed electrodes (ca. 10 mm far apart) show a lag time of 13.4 ± 4.5 s, and the diagonally placed electrodes (ca. 14 mm far apart) show a lag time of 53 ± 53 s. Also, this experiment confirms the presence of preferential paths, hindering conduction of electric potential between some electrodes and showing extreme variability between signal propagation speed.

**Figure 11:**
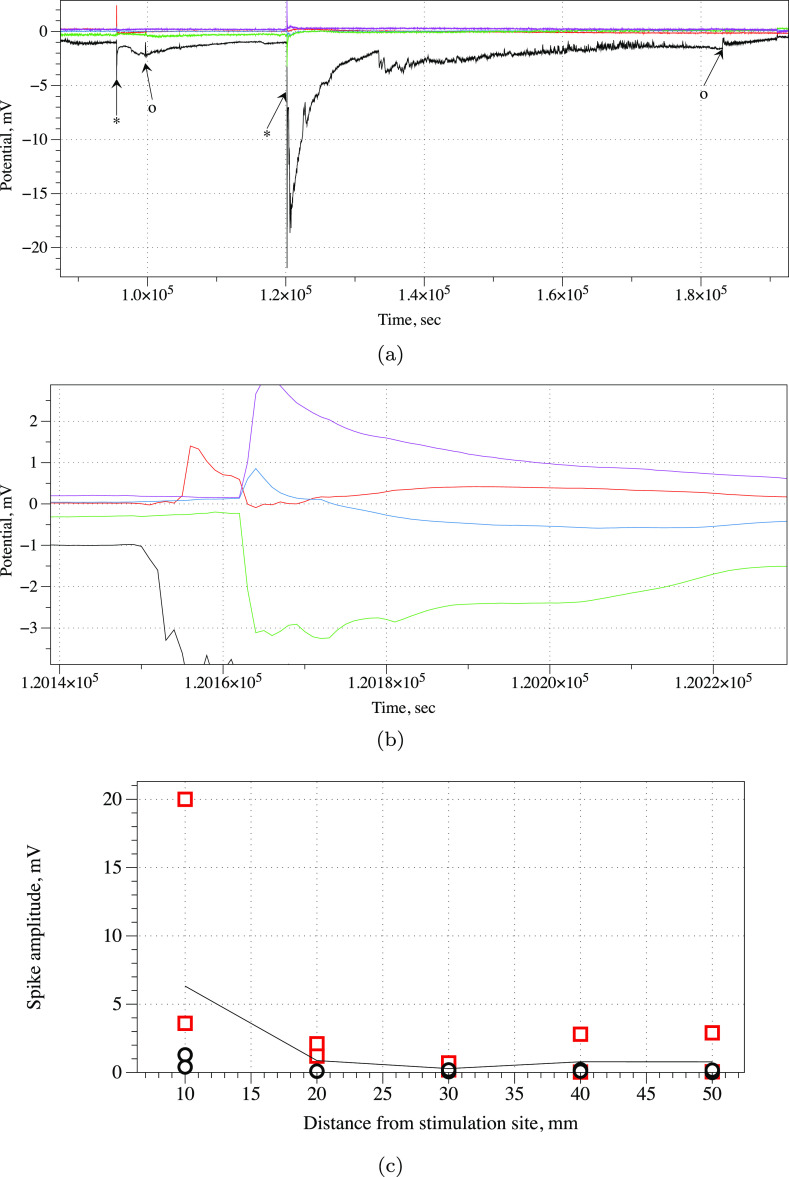
(a) Weight (nylon-coated still rod, 80 g weight, contact area with the *Acetobacter* pad was ca. 5 mm) applied (the moment of application is shown by ★) and removed (◦). The weight is applied near Ch1, black. Other channels in the line are Ch3 (red), Ch5 (blue), Ch7 (green), and Ch9 (magenta). (b) Zoomed Ch3-Ch9 at the moment of weight application. There is evidence of a distance-dependent response. (c) Dependence of the amplitudes of response spikes on a distance from the stimulation site. Red squares are amplitudes of responses to the load and black circles are responses to the unloading. The average amplitude (both load and unload) is shown by a solid line.

The assessment of the mechanical stimulus effect was measured through monitoring the spontaneous spiking activity, instead of using more conventional means of measuring a piezoresistive (or piezoelectric) response by injecting a current and measuring the voltage drop, or by applying a potential and measuring the current, or by monitoring the electrical oscillation frequency.^48^ The discrete nature of measurement electrodes necessarily introduces a loss of information, for some of the spiking produced in response to the physical stimuli might be lost. Exemplar responses to mechanical stimulation applied near channel 1 are shown in Figure 11a,b. The Acetobacter biofilm responds to applying and removing of the load with spikes of electrical potential. The average amplitude of the load application spike is 3.36 mV (σ = 4.74, median amplitude 1.42 mV), and the removal of the load has an average amplitude spike of 2.87 mV (σ = 4.13, median amplitude 1.45 mV). There is an average delay of 25 s in the responses between channels, which indicated that the action potential-like spike, originating at the load application site, travels along the *Acetobacter* biofilm. The average width of the spikes generated as a response to the application of the load is 106.8 s (σ = 56.45, median width 117.5 s), and a response to the removal of the load is 1478.4 s (σ = 3002.56, median width 145.5 s). The anomalously high standard deviation in the amplitudes and widths of the spikes can be explained by a distance-dependent response: spikes recorded on the electrode pairs closer to the mechanical stimulation site are larger than those further away from the stimulation site (Figure 11c). For what concerns the pressure effect, correlations are seen with an average lag time of 27.9 ± 24.7 s. The high standard deviation confirms that preferential paths exist in the colony.

## 4 Conclusions

In conclusion, we have analyzed the electronic and spiking properties of a living *Acetobacter aceti* biofilm, using advanced technologies which are currently employed in the characterization of condensed matter samples and devices. The dc properties of the biofilm put in evidence the fact that it behaves as an electrochemical battery, providing both an open-circuit voltage drop (always above 7 and below 25 mV) and a small but always nonzero short-circuit current (between 1.5 and 4 nA). The highest values of resistivity measured on the biofilm disk are found in the colony portion between the closer electrode couples, counter-intuitively, so that the preferential path network, invisible to the eye, provided by cellulose fibers to support the colony, takes a fundamental role in conveying the electrical signals. When performing impedance measurements, the outcome is that both differential resistance and the frequency parameter witnessing transition between capacitive and inductive responses are mostly influenced by the distance between the reference and active electrodes used for recordings, reaching the maximum at 180 and the minimum at 0. Pulse tomography allows us to reconstruct the preferential paths by collecting conduction properties radial-wise and inverting the information to plot a conductance map. Spiking activity shows a range of responses, featuring amplitudes on the order of 2 mV, widths of 200 s, spacing between spike trains of 500 s, and propagation speeds of 0.5 mm/s. Response to the application of weight produces spikes with average amplitudes higher than 3 mV with a delay of 100 s, while removal of the weight produces spikes with average amplitudes lower than 3 mV with a delay of 1500 s. The understanding achieved so far allows us to conclude that the living biofilm features non-trivial conduction properties and spontaneous spiking that can be correlated, for example, with pressure stimuli. Future research will prove the feasibility of a wearable living bacterial cellulose colonized glove with neuromorphic logics, able to transduce the spiking into useful information about finger bending and pressure stimuli.

## Notes

The raw/processed data required to reproduce these findings cannot be shared at this time due to technical or time limitations.
